# Supplementary material for: Overcoming gender inequality for climate resilient development
Source: Nat Commun. 2020 Dec 15;11:6261. doi: 10.1038/s41467-020-19856-w (PMC7738534; doi:10.1038/s41467-020-19856-w)
Supplement: Supplementary file 1 — Supplementary Information [file 41467_2020_19856_MOESM1_ESM.pdf]

Supplementary Material

Overcoming gender inequality for climate resilient development

Marina Andrijevic, Jesus Crespo Cuaresma, Tabea Lissner, Adelle Thomas, Carl-Friedrich Schleussner

	Dependent variable:			
	Gender Inequality Index			
	FE	FE	FE	OLS
	(1)	(2)	(3)	(4)
GDP per capita	−0.690***	−0.598***	−0.067**	−0.543***
	(0.031)	(0.031)	(0.028)	(0.029)
Gender gap in schooling		0.287***	0.162***	0.044**
		(0.034)	(0.025)	(0.021)
University education			−14.680***	−7.299***
			(0.737)	(0.428)
Observations	2,789	2,789	2,789	2,789
Multiple R^2	0.188	0.210	0.457	0.642
Adjusted R^2	0.140	0.160	0.423	0.642
Residual Std. Error	0.396 (df = 2629)	0.390 (df = 2628)	0.324 (df = 2627)	0.918 (df = 2785)
Note:				*p<0.1; **p<0.05; ***p<0.01

Supplementary Table 1: Regression results**.**
Robust standard errors are reported in parentheses. The coefficient estimates of our main specification and the basis for projections (Equation 2) in Methods are reported in column (3). Columns (1) and (2) show step-wise inclusion of the covariates in the primary specification. Column (4) shows the coefficient estimates of an OLS regression (without fixed effects terms).

Model validation

We assess the predictive ability of the variables used and the model employed using a simple validation exercise based on an out-of-sample predictive exercise. Using data spanning the period 2000-2005, we estimate an autoregressive model for our gender inequality variable, which serves as a benchmark to evaluate the (out-of-sample) predictive content of the information contained in the covariates of our specification. The autoregressive specification is given by

$$\mathrm{GII}^{*}_{\mathrm{i,t}} = \alpha_i + \vartheta \mathrm{GII}^{*}_{\mathrm{i,t-5}} + \varepsilon_{i,t},$$

implying that the dynamics of the gender inequality index can be explained by mean reverting dynamics around a country-specific equilibrium which is given by $\alpha_i/(1-\vartheta)$. Using this specification after estimating it for the period 2000-2005, we can obtain out-of-sample forecasts for all the countries in our sample for the year 2010. We also estimate a model that includes information about GDP per capita, education and the education gap, the three driving factors of gender inequality we consider in our main specification,

$$\mathrm{GII}^{*}_{\mathrm{i,t}} = \alpha_i + \vartheta \mathrm{GII}^{*}_{\mathrm{i,t-5}} + \beta_1 \ln \mathrm{GDPpc}_{\mathrm{i,t-5}} + \beta_2 \mathrm{education}_{\mathrm{i,t-5}} + \beta_3 \mathrm{educationgap}_{\mathrm{i,t-5}} + \varepsilon_{i,t},$$

where the covariates enter with a lag of five years to allow for five years-ahead out-of-sample predictions. After estimating this specification for the period 2000-2005, we can obtain predictions of the gender inequality index in 2010 for the countries in our sample based on a model that includes information on income and education dynamics. Expanding the set of in-sample observations to 2000-2006, we can obtain out-of-sample predictions for the year 2011 and repeating this exercise by expanding the sample used to estimate the model we can obtain 1202 five years-ahead forecasts spanning the period 2010-2017.

Supplementary Table 2 presents several standard measures of predictive error for the autoregressive (AR) specification and our model (MODEL) based on these forecasts. We compute (i) the mean squared forecast error (MSFE), which is the average of the squared deviations between realized and forecast values; (ii) the directional accuracy (DA) statistic, which gives the percentage of out-of-sample observations whose direction of change (increase or decrease) was correctly predicted, and (iii) the directional value (DV), which gives the average absolute value of the correctly predicted changes and should inform about whether the corresponding model fails at forecasting important changes in the target variable.

	**AR**	**MODEL**
RMSFE	0.306	0.283
DA	56.32%	68.64%
DV	0.152	0.207
Obs.	1202	1202

Supplementary Table 2: Out-of-sample validation exercise, model vs. benchmark AR specification

The results of the validation exercise based on the out-of-sample predictive ability of the model used give clear evidence that the covariates used in the model contain predictive information about future changes in the gender inequality index. In addition to reducing MSFE, the use of variables related to income, education and its distribution across genders increases directional accuracy very substantially, from around 56% correctly predicted changes to almost 69%. In addition, the changes which are forecast correctly are on average larger than those in the benchmark specification.

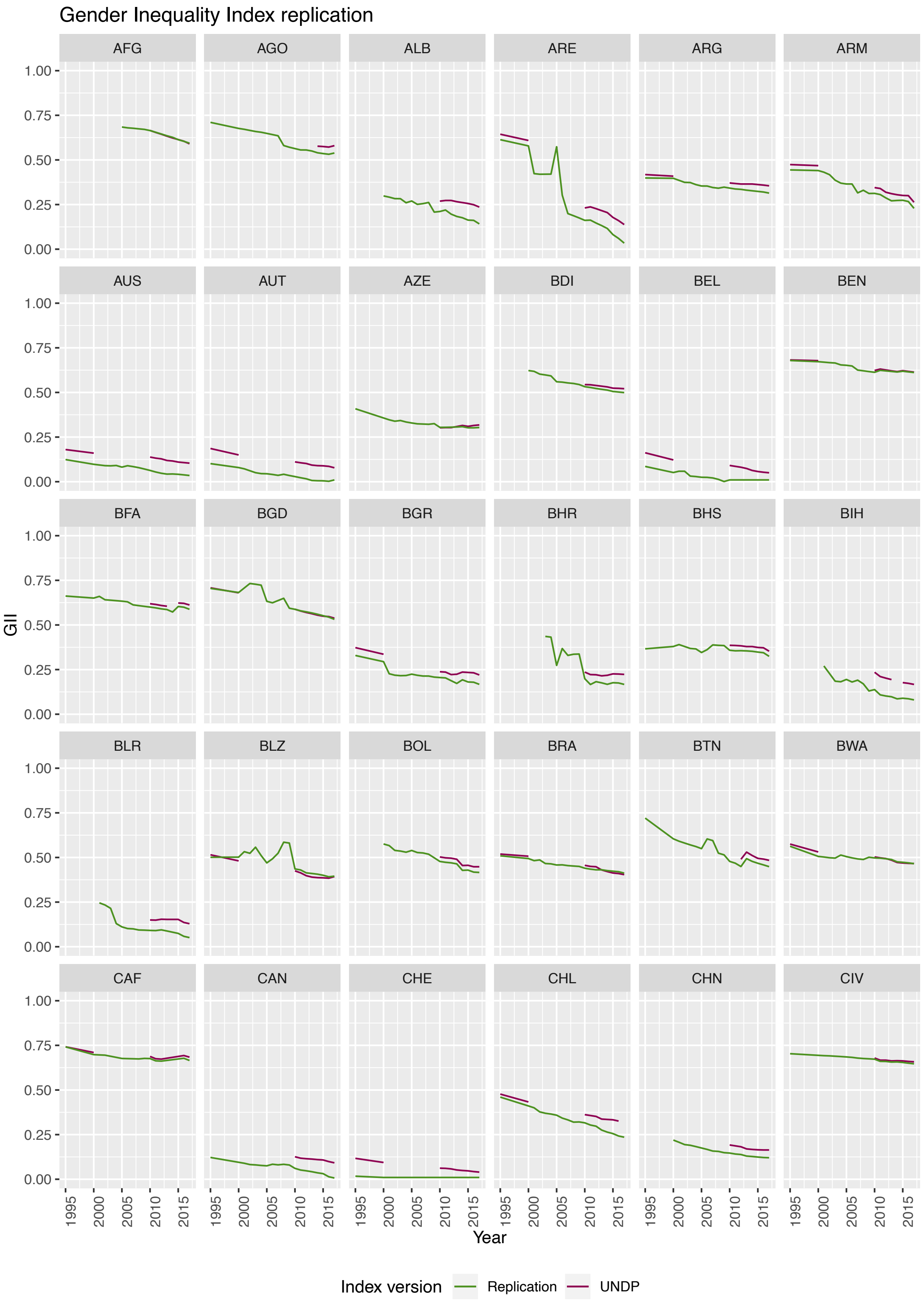
Gender Inequality Index replication
AFG
AGO
ALB
ARE
ARG
ARM
AUS
AUT
AZE
BDI
BEL
BEN
BFA
BGD
BGR
BHR
BHS
BIH
BLR
BLZ
BOL
BRA
BTN
BWA
CAF
CAN
CHE
CHL
CHN
CIV
GII
Year
Index version
Replication
UNDP

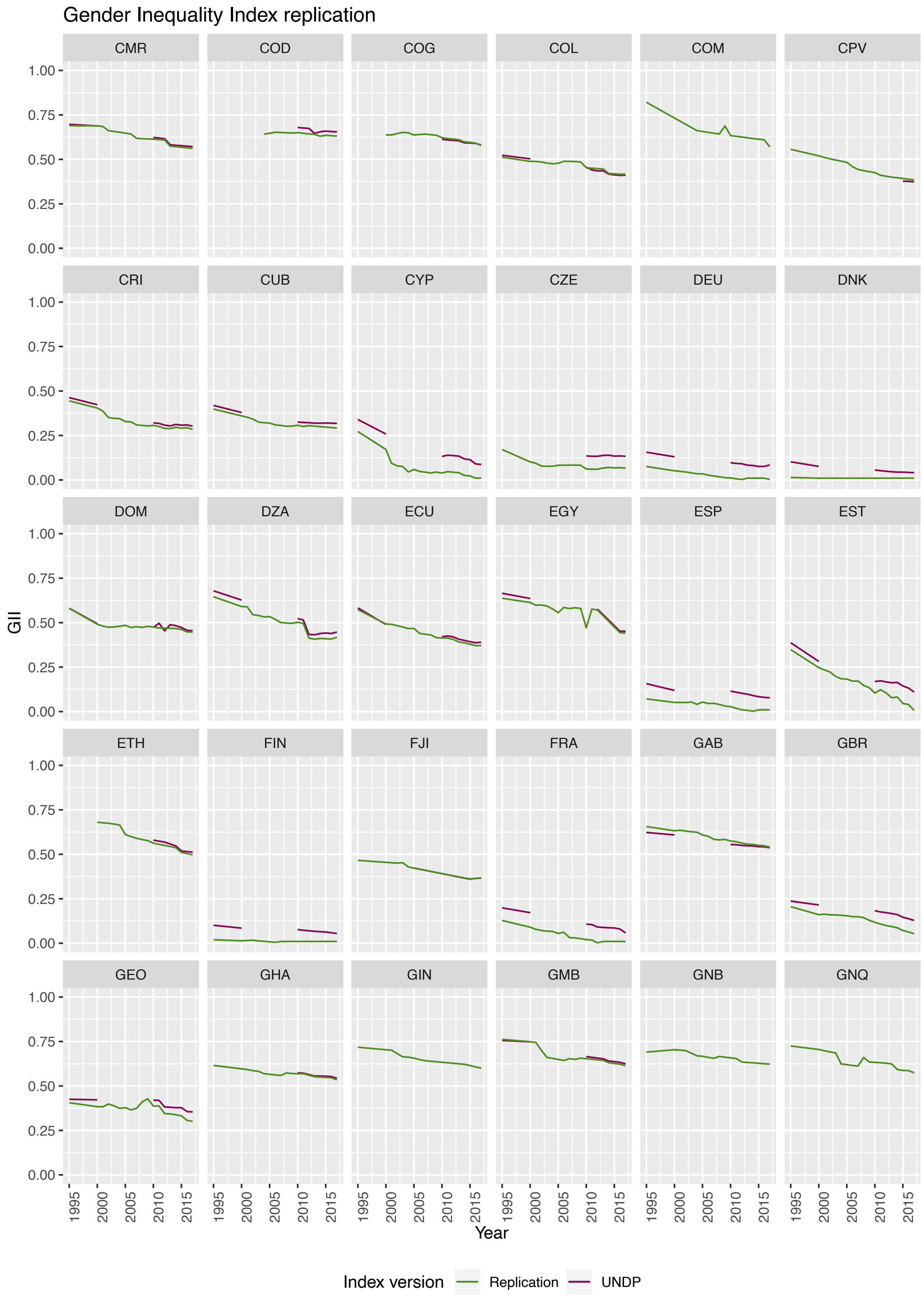

Gender Inequality Index replication
CMR
COD
COG
COL
COM
CPV
CRI
CUB
CYP
CZE
DEU
DNK
DOM
DZA
ECU
EGY
ESP
EST
ETH
FIN
FJI
FRA
GAB
GBR
GEO
GHA
GIN
GMB
GNB
GNQ
1.00
0.75
0.50
0.25
0.00
GII
1995
2000
2005
2010
2015
Year
Index version
Replication
UNDP

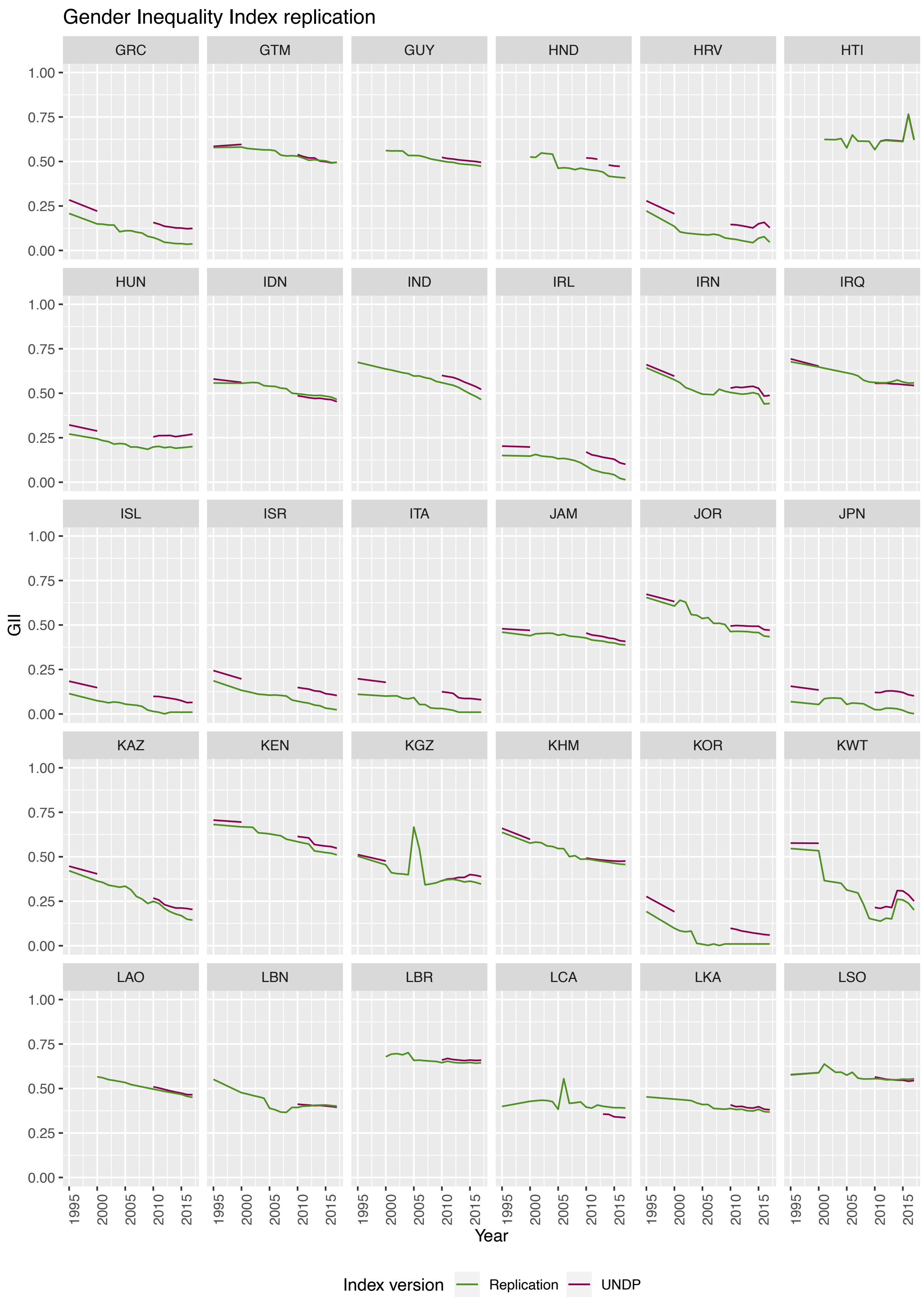

Gender Inequality Index replication
GRC
GTM
GUY
HND
HRV
HTI
HUN
IDN
IND
IRL
IRN
IRQ
ISL
ISR
ITA
JAM
JOR
JPN
KAZ
KEN
KGZ
KHM
KOR
KWT
LAO
LBN
LBR
LCA
LKA
LSO
1.00
0.75
0.50
0.25
0.00
GII
1995
2000
2005
2010
2015
Year
Index version
Replication
UNDP

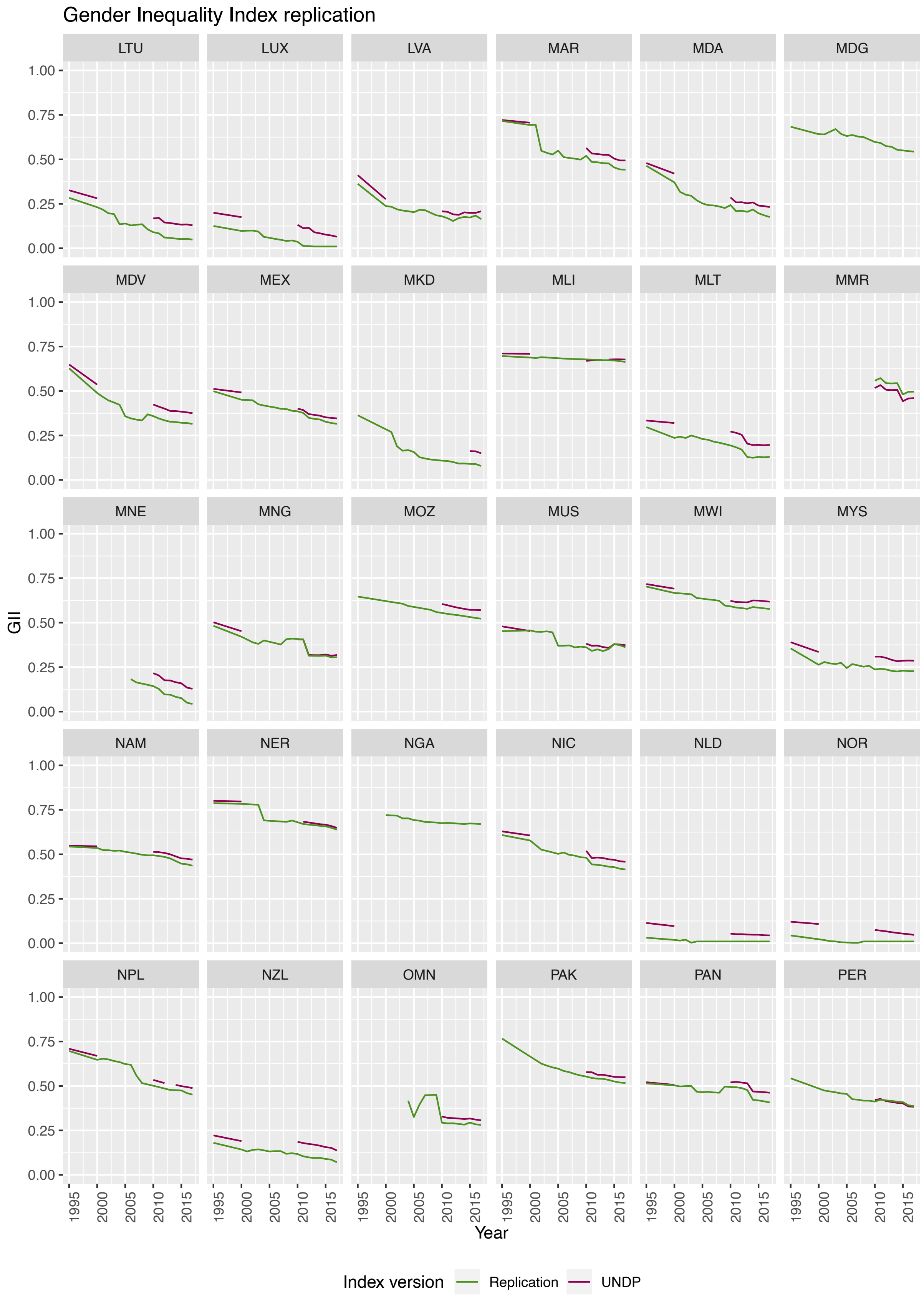

Gender Inequality Index replication
LTU
LUX
LVA
MAR
MDA
MDG
MDV
MEX
MKD
MLI
MLT
MMR
MNE
MNG
MOZ
MUS
MWI
MYS
NAM
NER
NGA
NIC
NLD
NOR
NPL
NZL
OMN
PAK
PAN
PER
GII
1.00
0.75
0.50
0.25
0.00
1995
2000
2005
2010
2015
Year
Index version
Replication
UNDP

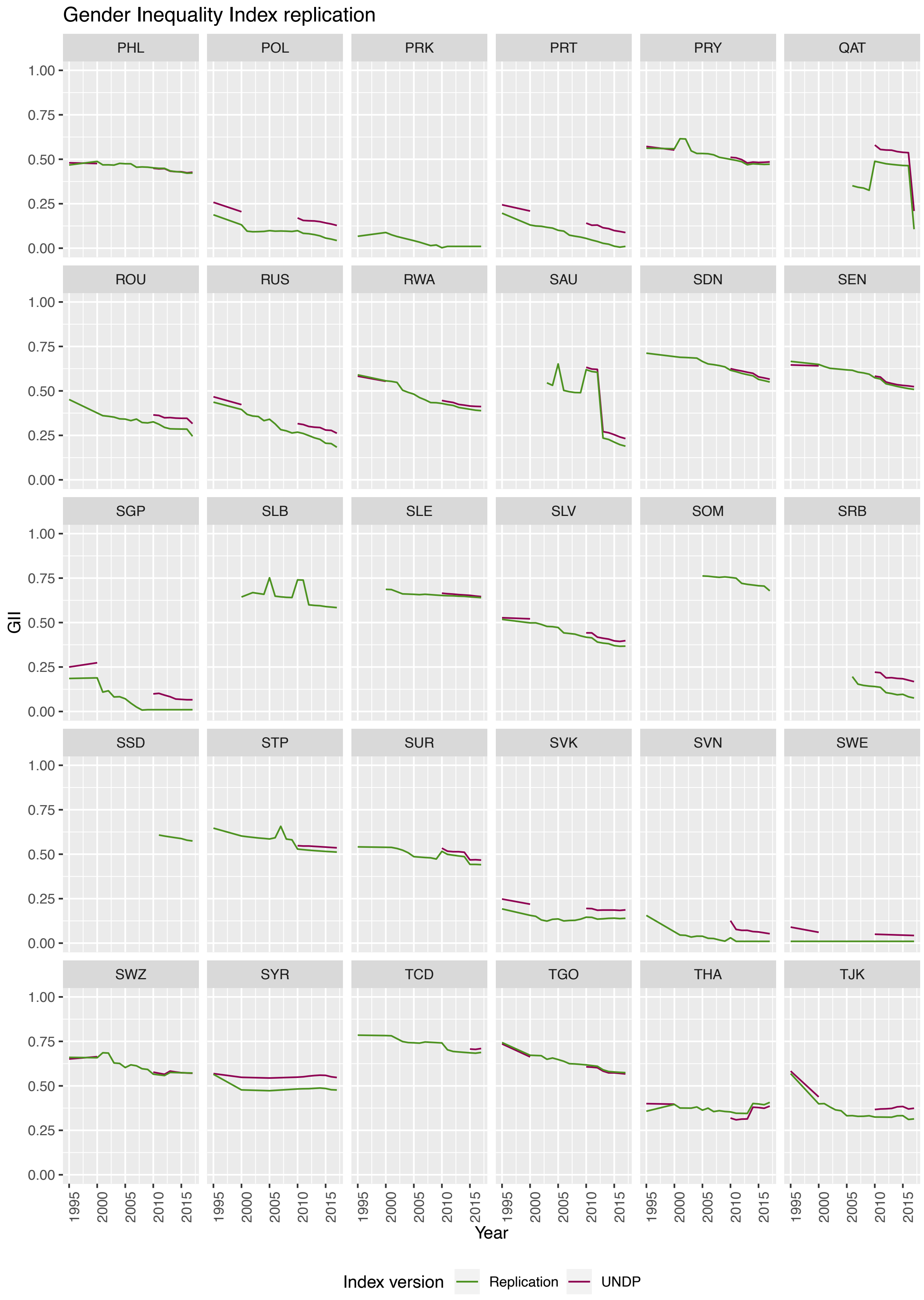

Gender Inequality Index replication
PHL
POL
PRK
PRT
PRY
QAT
ROU
RUS
RWA
SAU
SDN
SEN
SGP
SLB
SLE
SLV
SOM
SRB
SSD
STP
SUR
SVK
SVN
SWE
SWZ
SYR
TCD
TGO
THA
TJK
GII
1.00
0.75
0.50
0.25
0.00
1995
2000
2005
2010
2015
Year
Index version
Replication
UNDP

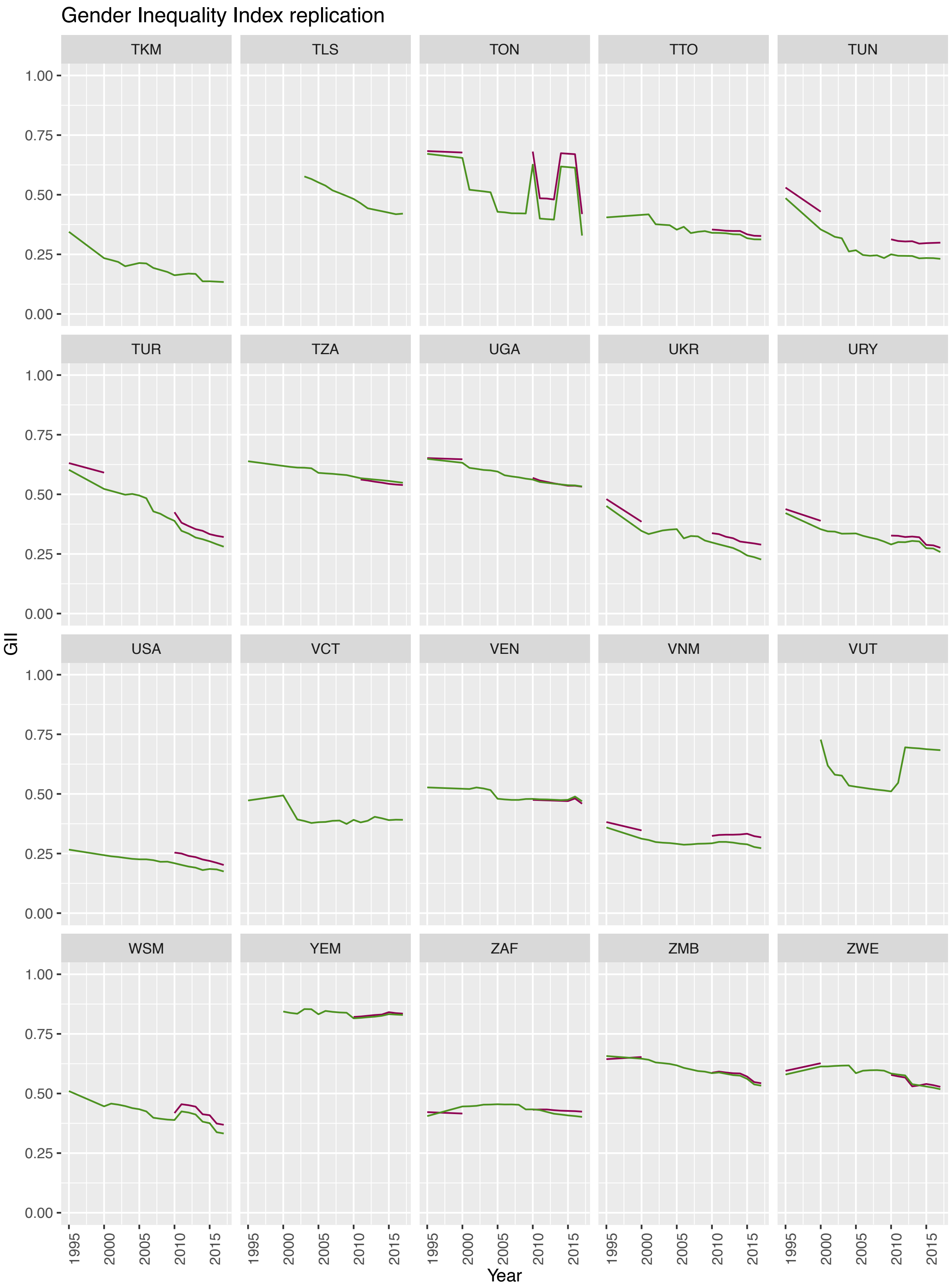

Supplementary Figure 1: Timeseries of the Gender Inequality Index (GII). The figure shows country-level values for the original index calculated by the UNDP and the authors' replication of the method.

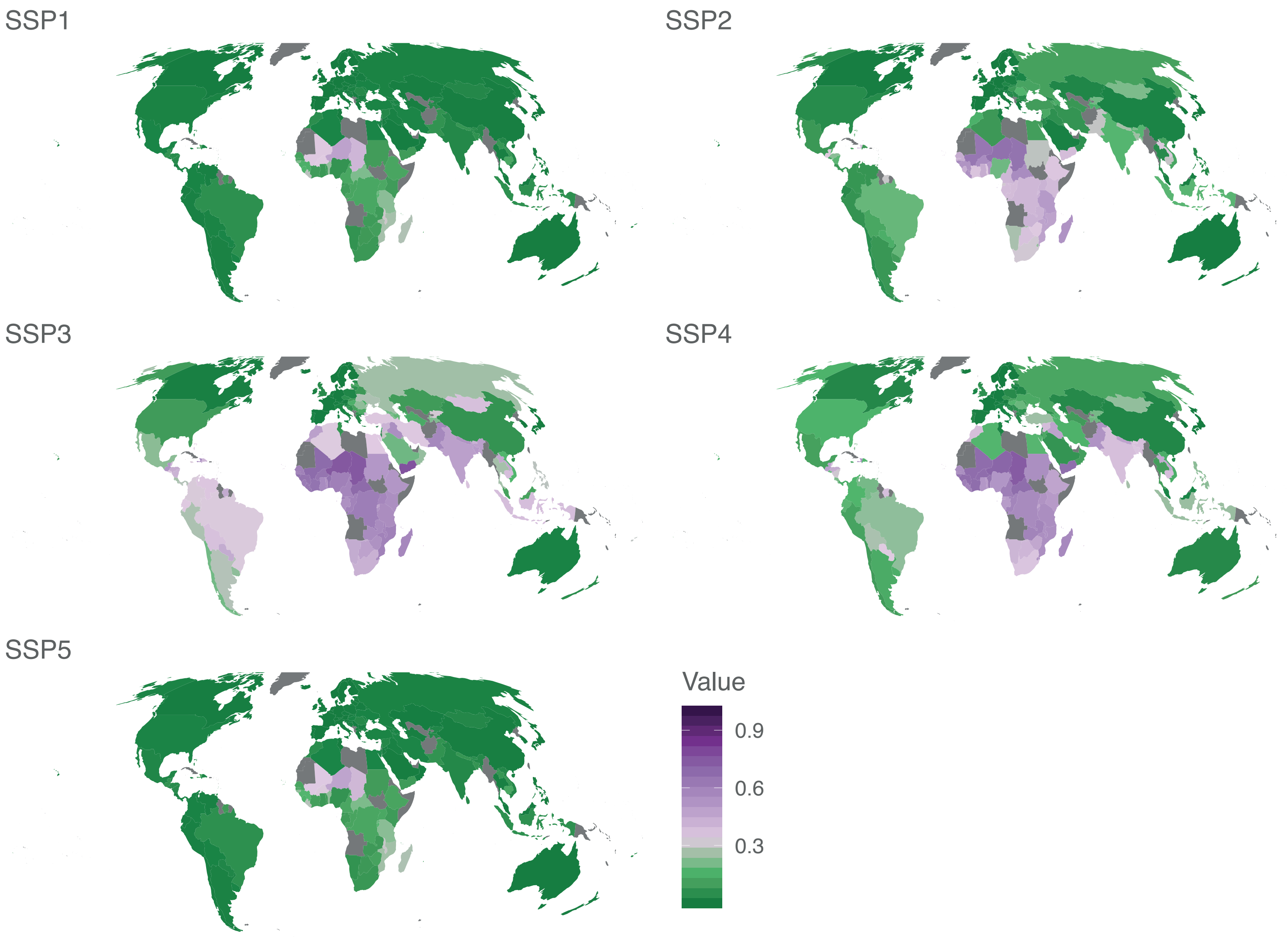

Supplementary Figure 2: GII projections for all SSPs in 2050.

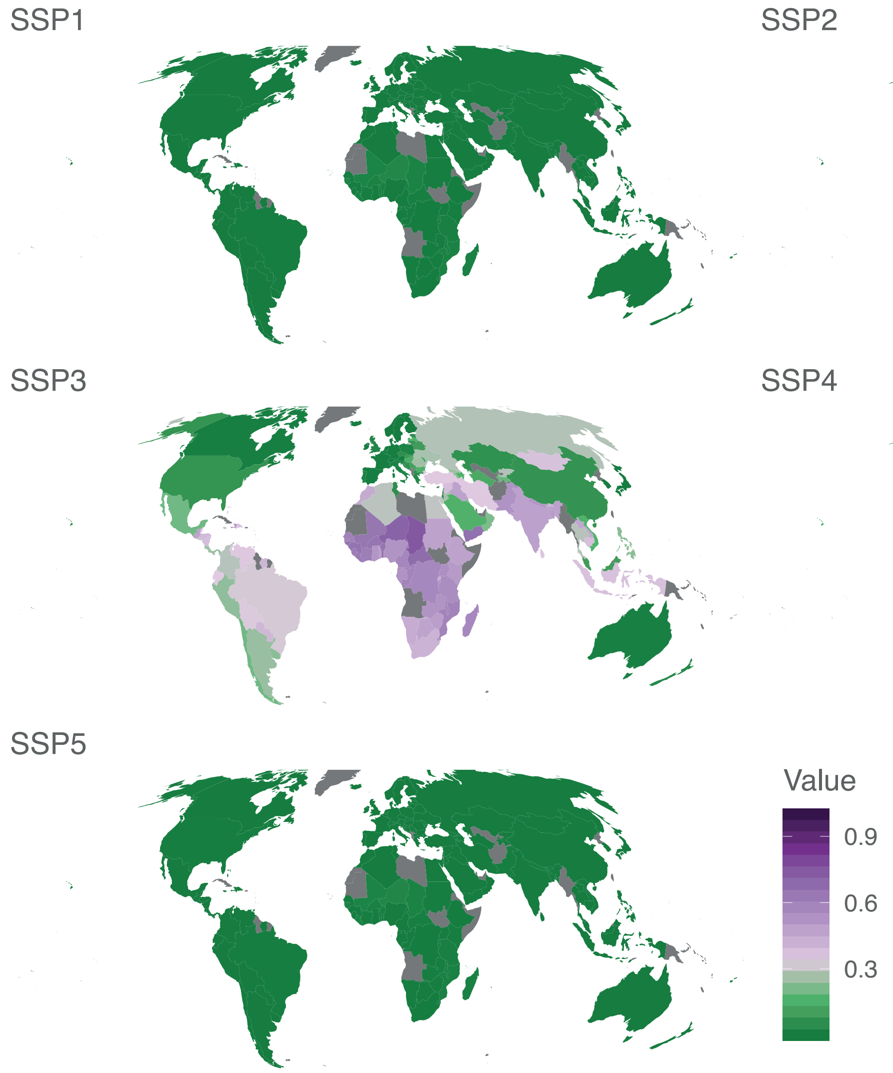

Supplementary Figure 3: GII projections for all SSPs in 2100.
