# Supplementary material for: Overcoming gender inequality for climate resilient development
Source: Nat Commun. 2020 Dec 15;11:6261. doi: 10.1038/s41467-020-19856-w (PMC7738534; doi:10.1038/s41467-020-19856-w)
Supplement: Supplementary file 3 — Reporting Summary [file 41467_2020_19856_MOESM3_ESM.pdf]

nature research

Corresponding author(s): Marina Andrijevic

Last updated by author(s): Oct 5, 2020

Reporting Summary

Nature Research wishes to improve the reproducibility of the work that we publish. This form provides structure for consistency and transparency in reporting. For further information on Nature Research policies, see Authors & Referees and the Editorial Policy Checklist .

Statistics

For all statistical analyses, confirm that the following items are present in the figure legend, table legend, main text, or Methods section.

n/a	Confirmed	
x		The exact sample size (*n*) for each experimental group/condition, given as a discrete number and unit of measurement
x		A statement on whether measurements were taken from distinct samples or whether the same sample was measured repeatedly
x		The statistical test(s) used AND whether they are one- or two-sided<br>*Only common tests should be described solely by name; describe more complex techniques in the Methods section.*
	x	A description of all covariates tested
x		A description of any assumptions or corrections, such as tests of normality and adjustment for multiple comparisons
	x	A full description of the statistical parameters including central tendency (e.g. means) or other basic estimates (e.g. regression coefficient) AND variation (e.g. standard deviation) or associated estimates of uncertainty (e.g. confidence intervals)
	x	For null hypothesis testing, the test statistic (e.g. *F*, *t*, *r*) with confidence intervals, effect sizes, degrees of freedom and *P* value noted<br>*Give P values as exact values whenever suitable.*
x		For Bayesian analysis, information on the choice of priors and Markov chain Monte Carlo settings
x		For hierarchical and complex designs, identification of the appropriate level for tests and full reporting of outcomes
x		Estimates of effect sizes (e.g. Cohen's *d*, Pearson's *r*), indicating how they were calculated

Our web collection on statistics for biologists contains articles on many of the points above.

Software and code

Policy information about availability of computer code

Data collection	This study used secondary data which was processed using R software version 1.3.1073.
Data analysis	All data analysis was performed in R software version 1.3.1073. The code and input data used in this analysis can be accessed at: https://github.com/marina-andrijevic/gender_equality2020

For manuscripts utilizing custom algorithms or software that are central to the research but not yet described in published literature, software must be made available to editors/reviewers. We strongly encourage code deposition in a community repository (e.g. GitHub). See the Nature Research guidelines for submitting code & software for further information.

Data

Policy information about availability of data

All manuscripts must include a data availability statement. This statement should provide the following information, where applicable:

- Accession codes, unique identifiers, or web links for publicly available datasets
- A list of figures that have associated raw data
- A description of any restrictions on data availability

Original Gender Inequality Index (GII) data is available through the UNDP website (http://hdr.undp.org/en/data). Data on maternal mortality ratio is available from UNICEF (https://data.unicef.org/topic/maternal-health/maternal-mortality/), and adolescent birth rates from WHO (https://www.who.int/gho/maternal_health/reproductive_health/adolescent_fertility/en/). Historical GDP was obtained from the Penn World Tables 7.0 (https://www.rug.nl/ggdc/productivity/pwt/pwt-releases/pwt-7.0) and projected values through the IIASA SSP database (https://tntcat.iiasa.ac.at/SspDb/). Data on educational attainment and gender gap in mean years of schooling is accessible through the Data Explorer of the Wittgenstein Centre for Demography and Global Human Capital (http://dataexplorer.wittgensteincentre.org/wcde-v2/). The code for the replicating the GII reconstruction and the projections exercise can be found at: https://github.com/marina-andrijevic/gender_equality2020

Field-specific reporting

Please select the one below that is the best fit for your research. If you are not sure, read the appropriate sections before making your selection.

☐ Life sciences ☐ Behavioural & social sciences ☒ Ecological, evolutionary & environmental sciences

For a reference copy of the document with all sections, see nature.com/documents/nr-reporting-summary-flat.pdf

Ecological, evolutionary & environmental sciences study design

All studies must disclose on these points even when the disclosure is negative.

Study description	This study highlights the importance of accounting for gender inequality in climate change research and policy. It analyzes the historical response function of a country-level indicator of gender inequality in relation to country-level measures of income, education and gender gap in education, and based on the coefficient estimates obtained, projects the indicator of gender inequality forward along five different scenarios of socio-economic development.
Research sample	The sample contains between 126 and 159 countries (depending on the year).
Sampling strategy	The sample is determined by data availability on the country level.
Data collection	This study used indicators from open-access databases (see detailed description above in the Data section)
Timing and spatial scale	The data is yearly (1995 - 2017 for the observed period, and 2020 - 2100 for the projections period) and on the country-level.
Data exclusions	No data was excluded from the analysis.
Reproducibility	This study does not involve experimental methods. All statistical analyses can be replicated with the code provided at: https://github.com/marina-andrijevic/gender_equality2020
Randomization	This study does not involve experimental methods. The whole sample is included in the statistical analysis.
Blinding	This study does not involve experimental methods, hence blinding is not relevant.

Did the study involve field work? ☐ Yes ☒ No

Reporting for specific materials, systems and methods

We require information from authors about some types of materials, experimental systems and methods used in many studies. Here, indicate whether each material, system or method listed is relevant to your study. If you are not sure if a list item applies to your research, read the appropriate section before selecting a response.

Materials & experimental systems

n/a	Involved in the study	
☒	☐	Antibodies
☒	☐	Eukaryotic cell lines
☒	☐	Palaeontology
☒	☐	Animals and other organisms
☒	☐	Human research participants
☒	☐	Clinical data

Methods

n/a	Involved in the study	
☒	☐	ChIP-seq
☒	☐	Flow cytometry
☒	☐	MRI-based neuroimaging
